# A Systematic Review of the Abdominal Surgeon’s Personality: Exploring Common Traits in Western Populations

**DOI:** 10.3390/bs11010002

**Published:** 2020-12-26

**Authors:** Carly Nichola Bisset, Tracey McKee, Mary Cawley, Elliot Tilling, Susan Joan Moug

**Affiliations:** 1 Department of Medical Education, University of Aberdeen, Aberdeen AB24 3FX, UK; 2 Department of General Surgery, Royal Alexandra Hospital, Paisley PA2 9PN, UK; elliot.tilling@nhs.net (E.T.); susan.moug@ggc.scot.nhs.uk (S.J.M.); 3 NHS Greater Glasgow and Clyde Knowledge Services, Glasgow G3 8BW, UK; tracey.mckee@ggc.scot.nhs.uk; 4 West of Scotland Renal & Transplant Unit, Queen Elizabeth University Hospital, Glasgow G51 4TF, UK; Mary.Cawley@ggc.scot.nhs.uk; 5 College of Medical, Veterinary & Life Sciences, University of Glasgow, Glasgow G12 8QQ, UK

**Keywords:** systematic review, personality traits, Big Five, surgeon personality, decision-making

## Abstract

The personality traits commonly seen in abdominal surgeons remains undefined, and its potential influence on decision-making and patient outcomes underexplored. This systematic review identified studies on abdominal surgeons who had undergone validated personality testing, with assessment of decision-making and post-operative patient outcomes. The study protocol was registered on PROSPERO (University of York, UK (CRD42019151375)). MEDLINE, Embase, PsycInfo and Cochrane Library databases were searched using the keywords: surgeon; surgeon personality; outcomes. All study designs were accepted including adult visceral surgeons published in English. Five articles from 3056 abstracts met our inclusion criteria and one article was identified from hand searches with two reviewers screening studies. Bias was assessed using the Newcastle-Ottawa scale. Six studies included 386 surgeons. Studies assessing personality using the Five Factor Model (four studies, 329 surgeons) demonstrated higher levels of conscientiousness (self-discipline, thoughtfulness), extraversion (sociability, emotional expression) and openness (creative, conventional) in surgeons versus population norms. Surgeon characterisation of agreeableness and emotional stability was less clear, with studies reporting mixed results. Post-operative outcomes were reported by only one study. Further exploration of the influence of surgeon personality and its influence on decision-making is necessary to deliver patient-centred care and targeted non-technical skills training for surgeons.

## 1. Introduction

Personality is largely regarded as the “pattern of thoughts, feelings and social behaviours consistently exhibited over time… influencing one’s expectations, self-perception, values and attitudes” [1]. Despite common misconceptions that personality is a fixed entity, personality has been shown to change with age [2], varies with gender [3], and may be influenced by higher education and life experiences in response to one’s environment [4]. Personality has been found to influence critical decision-making in high-risk vocations such as space exploration and the military [5,6,7]. Consequently, attention has turned to the healthcare setting, where complex decision-making bears implications for the clinician, the patient, and their wider support network. Whilst there is clear evidence that patient personality influences post-operative outcomes [8,9,10], the study of surgeon personality, to date, has largely explored the idea of targeted recruitment to minimise attrition [11,12], with limited exploration of the role of surgeon personality on decision-making and, subsequently, patient outcomes. Such decisions may include but are not limited to: the decision to operate versus non-operative management, pre-operative procedure planning, intra-operative changes in approach and post-operative management, including the response to complications [13].

The concept of ‘high’ or ‘low’ risk in surgery is complex, with no consensus definition in existence. Higher risk may be associated with specific patient factors such as comorbidities, or surgery-specific factors such as the intended operation [14], or higher incidence of post-operative mortality above a pre-defined threshold [15,16]. Lesser known is the influence of the surgeon’s personality on risk perception when considering the perceived risks or benefits of surgical intervention specific to each field. For example, a high-risk decision (accounting for both patient and procedure-specific risk factors) in vascular surgery (e.g., aortic aneurysm repair) is unlikely to be comparable to a high-risk decision in orthopaedic surgery (e.g., spinal laminectomy). This review is therefore limited to the study of the abdominal (visceral) surgeon’s personality, due to similar training pathways and similarities found in high-risk decisions, such as gastrointestinal anastomoses.

In abdominal surgery, several studies on laparoscopic cholecystectomy have described how surgeon personality influences risk perception and cognitive bias (errors of thought), including plan continuation (i.e., where there is unwise tendency to commit to a failing plan, for example, misidentification of anatomy despite visual cues) [17,18]. Despite these cues, management errors still occur, leading to misdiagnosis (irrespective of surgeon experience) and patient harm including bile duct injury [17]. Personality may also influence stoma formation rates and anastomotic practice in colorectal surgery, as demonstrated by the Edinburgh Delphi [19]. The Five Factor Model [20] (consisting of (1) agreeableness—degree of cooperation, easy-going; (2) conscientiousness—self-discipline, thoughtfulness; (3) emotional stability—neuroticism or even temperament; (4) extraversion—sociability, emotional expression and (5) openness to experience—creative or conventional), was used to define the personality traits of fifty colorectal surgeons [19]. This study found that personality influenced anastomotic decision-making following recent critique from a colleague (high openness) or when working with an unfamiliar anaesthetist (low openness). Therefore preliminary evidence exists to suggest that personality is influential in decision-making in both non-medical and medical fields, however, it is currently unclear what the exact role of the surgeon’s personality has on decision-making in abdominal surgery.

The primary objective of this systematic review was to summarise the existing literature to determine if common personality traits are present amongst abdominal surgeons, and determine if these influence decision-making. Our secondary objective was to determine if abdominal surgeon personality influences post-operative outcomes in adult populations. 

We hypothesised that while the current literature regarding abdominal surgeon personality would be somewhat limited, a review of what has been established previously was necessary to design a novel study assessing surgeon personality and anastomotic decision-making by the same authors (The Plato Project—an international cohort study of colorectal surgeons, performing personality testing and exploring risk perception in response to hypothetical clinical scenarios).

## 2. Materials and Methods

### 2.1. Protocol and Registration

Our systematic review was first registered on PROSPERO (University of York, UK) in September 2019 (CRD42019151375), and the full protocol published in February 2020 (PMID 32019819) [21]. No deviations from the protocol occurred. PRISMA guidelines were followed for reporting [22].

### 2.2. Eligibility Criteria

The group of interest was adult abdominal (general) surgeons who had undergone validated personality testing (e.g., Five Factor Model, Life Styles Inventory). Primary outcomes included trait predominance in abdominal surgeon groups and decision-making outcomes if reported. Secondary outcomes included post-operative patient outcomes following surgery.

The search was limited to studies published in the English language, human subjects and surgeon personality/behaviour only (not patient personality), with no limitation on the date of publication. Studies involving other abdominal specialties such as gynaecology, vascular and urology were excluded due to differences in operative risk associated with operations in these fields in comparison to gastrointestinal surgery. Paediatric surgery was excluded due to perceived differences in risk perception between adult and paediatric surgeons. We hypothesise that given the inherent emotive nature of paediatric surgery, the likelihood of pursuing an aggressive surgical approach to the critically unwell child may be more likely than (for example) the critically unwell older adult.

### 2.3. Information Sources

A literature search was performed on 1st October 2019 using database subject headings and key words relating to “abdominal/general surgeons”, “personality”, “post-operative outcomes”, and “decision-making”. The following databases were used: MEDLINE (R) ALL (OVID interface, 1946 onwards), Embase and Embase Classic (OVID interface, 1947 onwards), PsycInfo (OVID interface 1806 onwards) and Cochrane Library (Wiley interface, current issue). Where clarification was required, the original study authors were contacted.

### 2.4. Search

The databases were searched by a Health Librarian, with prior systematic review experience, in consultation with the review team (Supplementary Materials) [22]. The final draft MEDLINE strategy was peer reviewed by another Health Librarian not involved in the review. The strategy was adapted to each database to take account of differences in controlled vocabulary and search functionality. To ensure literature saturation, reference lists of included studies were reviewed to include relevant studies.

### 2.5. Study Selection and Data Collection

Abstracts from the initial search were added to a RefWorks database. A Microsoft Excel spreadsheet was used by the authors to record the outcomes of the screening process. Two reviewers screened articles (2 of C.B., E.T., S.M.), with a third, impartial reviewer (M.C.) making the final decision if there was not consensus. Where there was uncertainty regarding eligibility, a third author (M.C.) was invited to discuss each case to reach a conclusion with rationale recorded. Where data was missing, contact was made with the original study authors.

### 2.6. Data Items

Data extraction from full text articles was recorded using a spreadsheet and is reported in Table 1.

### 2.7. Risk of Bias in Individual Studies

The Newcastle-Ottawa scale was applied to assess the risk of bias, as each of the included studies was either a case control or cohort study [23]. Each domain of the scale was graded as per the universal marking, in a “star” format to act as a visual tool regarding quality. The collaborating authors also assessed the risk of bias within each study (high, medium, or low risk of bias).

### 2.8. Summary Measures

The principal summary measures assessed differences in mean values of personality domain scores, to enable comparisons with population norms or physician groups.

### 2.9. Synthesis of Results and Risk of Bias Across Studies

A systematic narrative synthesis is provided in our results, with further information presented via text and tables. The narrative synthesis explores the relationship and findings between studies, in accordance with guidelines from the Centre for Reviews and Dissemination [24]. 

Clinical heterogeneity was tested where possible to include participant demographics such as age and gender. Despite defined key words to include abdominal surgeons, heterogeneity was present, therefore, it was not appropriate to perform quantitative synthesis in the form of meta-analysis.

## 3. Results

### 3.1. Study Selection

**Figure 1:**
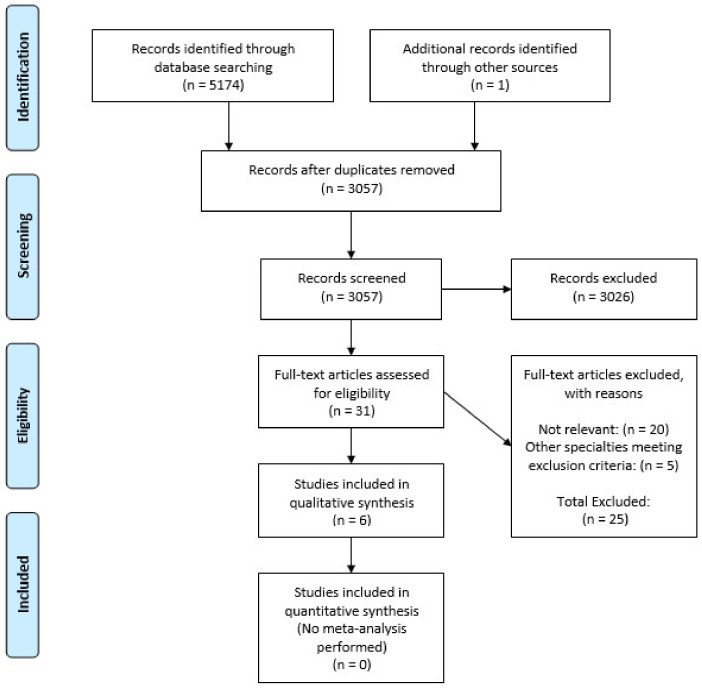
PRISMA 2009 flow diagram.

Using the search criteria (Supplementary Materials), a total of 5174 articles were identified. After adjusting for duplicates, 3056 remained with a further 3026 excluded as they did not meet the inclusion criteria. After review of the 30 full text articles, a further 25 studies were excluded. An additional study was identified by checking the references of included articles (Figure 1 and Supplementary Materials).

### 3.2. Study Characteristics and Results.

Six studies had the following data extracted: sample size, study type, single vs. multi-institution, country, and validated personality test used as well as secondary outcomes (Table 2 and Table 3) [19,25,26,27,28,29]. The total number of responding participants for inclusion was 386, with sample sizes ranging from 22–179 participants per study. In all studies, the primary outcome was assessed as a raw mean in personality domain, with peri-operative decision-making specifically reported by two studies [19,25]. Gender differences in personality were described by three studies. Secondary outcomes were reported by a single study [25]. All six studies were performed in either the UK (50%) or the USA (50%). Population norms are therefore reported using the general population comparisons reported within each study and are thus representative of that country [30,31,32,33]. 

### 3.3. Risk of Bias Within Studies

Individual assessment of the risk of bias within each study is summarised in Table 4 using the Newcastle-Ottawa Scale. Using this scale, half of the included studies had a ‘low’ risk of bias, two were ‘medium’ risk and one had a ‘high’ risk of bias. No study self-reported their own risk of bias. 

### 3.4. Risk of Bias Across Studies

Small-study effects may be seen in the included articles, increasing the likelihood of sampling bias within this review. Each study used a different validated personality index tool, meaning that subgroup analysis is not possible to compare the results drawn from each index. Not all studies reported the influence of surgeon experience, age or gender in relation to personality traits. To report publication bias, we assessed articles per the Outcome Reporting Bias in Trials (ORBIT) classification (Table 4) [34]. 

### 3.5. Synthesis of Results

Four out of six studies (66.7%) used a version of the Five Factor Model to assess personality within their surgeon population. One study used the Life Styles Inventory to describe leadership style and communication strategy, while a further study used the Eysenck Personality Questionnaire (describing degree of extraversion and neuroticism only), alongside the lesser used Edinburgh Six Factor Model (assessing self-rated intelligence; conscientiousness; emotional stability; extraversion; will and affection) [35] (Table 3).

### 3.6. Narrative Synthesis

#### 3.6.1. Surgeon Personality

Studies using the Five Factor Model demonstrated that surgeons displayed significantly higher levels of the following traits when compared to population norms: conscientiousness (four studies), extraversion (three studies) and openness (three studies). Surgeon characterisation of agreeableness and emotional stability/neuroticism were less clear, with studies finding mixed results (Table 3). The only study comparing surgeons to another clinical population (physicians) found that while all doctors scored more highly in extraversion, conscientiousness and agreeableness than the general population, surgeons generally displayed higher levels of extraversion and conscientiousness with lower levels of agreeableness in comparison to physicians [27].

#### 3.6.2. Personality Differences amongst Abdominal Surgeons: The Influence of Surgeon Experience

Two studies examined the effect of surgeon experience on personality. Drosdeck et al. compared the personality traits of trainee surgeons with experienced general surgeons in a single institution, finding higher levels of agreeableness in trainees [27]. In Whitaker et al.’s study of members of the Royal College of Surgeons of England, older surgeons in this cohort displayed higher levels of neuroticism compared to younger surgeons [29]. As only two studies examined differences in surgeon age as a surrogate for experience, there is insufficient evidence from this review to conclude or refute that the older generation of surgeons may have differing personality traits to younger surgeons.

#### 3.6.3. Personality Differences amongst Abdominal Surgeons: The Influence of Surgeon Gender

Three studies reported gender as part of their personality analysis. While McGreevy et al. did not demonstrate significant gender differences in personality types in surgeon trainees, they did find that female trainees may display higher levels of expressed traits, particularly openness and extraversion [28]. These findings are similar to Whitaker et al.’s study, where female surgeons scored even higher in extraversion and agreeableness fields than their male counterparts, who already scored higher in these traits than population norms [29]. However, Deary et al. did not demonstrate any appreciable differences in personality between male and female surgeons or male and female surgical trainees. While most evidence suggests that female and male surgeon personalities are similar, female surgeons may display higher degrees of these traits, in particular: openness, extraversion and agreeableness.

#### 3.6.4. Personality and Operative Decision-Making

Each study was assessed for whether or not a peri-operative decision had been reported in relation to surgeon personality. Only two studies directly commented upon peri-operative decision-making, which was defined as either: alteration in intended operation or altered decision-making post-operatively, such as in the occurrence of complications. In both studies, the decision-makers were consultant gastrointestinal surgeons. Given the small number of studies reporting altered decision-making, direct comparison was not possible and, therefore, thematic analysis not feasible. We therefore present a short summary of the main findings below.

Moug et al. assessed the personality traits and decision-making of 50 consultant surgeon members of the Association of Coloproctology of Great Britain and Ireland (ACPGBI) [19]. Most participants were male (86%) and UK & Ireland-based (82%). Surgeons in this cohort scored highly in emotional stability (5.4 vs. 4.8) and conscientiousness (6.1 vs. 5.4) compared to population norms. Moug et al. also demonstrated that particular personality traits influenced the colorectal surgeon’s decision to primarily anastomose or form a defunctioning stoma (e.g., high levels of openness following recent critique at a morbidity and mortality meeting; or high levels of alexithymia or low openness when working with an unfamiliar anaesthetist are more likely to form a defunctioning stoma). Tentative evidence therefore suggests that the colorectal surgeon’s personality influences the decision to form stomas or anastomose in complex cases, however further exploration of this is required to validate this study’s findings.

Shubeck et al. analysed 35 bariatric surgeons (89% male) based in Michigan, USA, using the Life Styles Inventory to describe the leadership style and decision-making behaviour of surgeons, with inferences made on their personality [25]. The authors found that although a single dominant leadership style did not exist, some leadership styles were associated with poorer post-operative outcomes (for example, low passive/defensive leadership styles).

#### 3.6.5. Secondary Outcomes: Surgeon Personality and Patient Outcomes

Shubeck et al.’s study was the only one to report on our secondary outcomes. Adverse events were defined by the study as including any of the following complications within 30 days of bariatric surgery: cardiopulmonary complications, renal failure, haemorrhage, surgical site infection, wound complication, venous thromboembolism, anastomotic leakage, bowel obstruction, hospital-related infection and death. The authors concluded that low levels of passive/defensive leadership style were associated with the highest rates of adverse events following bariatric surgery (18.7%), and proposed that this was due to discouragement of initiative and creativity (‘thinking outside of the box’) [25].

## 4. Discussion

Abdominal surgeons routinely make complex decisions that affect both the surgical procedure and ongoing care of their patients. This systematic review found only a small number of studies which examine the abdominal surgeon’s personality, with variability in reporting measures. With evidence that personality influences decision-making in other fields requiring critical decisions, this review supports the need for urgent future work into defining the influence of personality on cognitive bias and complex decision-making, and consensus usage of the Five Factor Model to reduce study heterogeneity.

The traits expressed in this review by abdominal surgeons (high levels of conscientiousness, extraversion and openness) provisionally appear to be similar to surgeon groups from other specialties [36,37], however, the literature regarding other specialties is extremely limited, with, for example, only one study examining the personalities of cardiac surgeons [36]. These similarities may be partly explained by ‘herd mentality’, where surgical programme applicants identify with traits displayed by their role models, thus perpetuating a similar ‘type’ or culture of similar personalities [38]. The common surgical traits described in this review may be considered advantageous in establishing rapport with patients and, perhaps, contradicts the ‘surgeon stereotype’ of rudeness, aggression and being difficult to work with [39]. Patients have previously indicated that they believe that surgeon personality influences surgical decision-making (including informed consent and communication of treatment options) as well as the surgeon’s response to post-operative complications [40]. Whilst we do not advocate personality testing as a recruitment strategy for trainee selection, the authors hypothesise that periodic personality testing of surgeons throughout their career (given that personality changes with age and experience) may facilitate continuing professional development via multiple methods, for example, targeted non-technical skills training for surgeons, and also aid identification of surgeons at risk of work-related stress or burnout with timely occupational health interventions [41]. 

It is possible that the surgeon’s personality and tolerance of risk influences decision-making, particularly in complex circumstances which may change with experience [38]. The influence of personality is likely to be multifactorial and includes: interpersonal skills with colleagues (including incivility) [42], management of emotions (particularly under stress) and comfort with risk [38,43,44]. Whilst personality is a complex concept, it is invariably linked but distinct to emotional intelligence [45], grit [46] and heuristics [43]. It has been reported previously that older surgeons are less likely to form defunctioning stomas and take more risks than younger surgeons; the inverse of the normal population [43,44]. This may be because older surgeons are more comfortable with risk or that patterns in stoma formation training have changed. The influence of surgeon age and/or experience, therefore, merits further investigation.

The investigation of personality and cognitive bias has been explored in greater detail in other medical specialties. For example, a systematic review of physician personality and cognitive bias demonstrated that overconfidence, tolerance to risk and confirmation bias influenced decision- making [47], however, only two studies explored physician personality in relation to patient outcomes. In obstetrics, higher tolerance of ambiguity (usually associated with high levels of emotional stability and openness) [48] was associated with increased risk of postpartum haemorrhage (9.7% vs. 6.5%, *p* = 0.004) [49]. Optimal performance in emergency retrieval medicine was seen in team members who displayed higher levels of emotional intelligence, higher levels of conscientiousness and openness [50].

In high-risk vocations outwith medicine (such as spaceflight and the military, where human factor parallels are often drawn), personality trait testing is well established. For example, the European Space Agency uses personality testing as a strong indicator of psychological recommendation for mission testing in astronaut recruitment [5]. Successful recruits consistently have lower levels of neuroticism (i.e., high emotional stability), which has been found to be beneficial in other astronaut cohorts and in submariners [6,51]. In a study of 531 US military pilots, Chidester et al. found that personality traits were potentially modifiable and could improve the likelihood of safe missions, where pilots showed willingness to change before and after appropriate decision-making training (i.e., possessed higher levels of openness) [7]. Chidester et al.’s study found that subjects with higher rates of positive instrumental and expressive traits benefited most from simulation training [7]. This raises the possibility that simulation training in surgery may be modified based upon the learner’s personality traits. This is a novel concept which merits further investigation, as tailoring non-technical skills training with attention to the surgeon’s personality could be more effective than the current ‘one size fits all’ approach to continuing professional development exercises and interventions [52,53].

### Limitations

We acknowledge that this systematic review only included a small number of studies of varying quality, with a small number of participants per study. Abdominal surgeons were selected as they share common training pathways and differences in personality, and decision-making may exist between surgical specialties. However, heterogeneity remains within the abdominal surgeon cohort, with the inclusion of trainees, bariatric surgeons, colorectal surgeons and general surgeons. Inherent gender bias also exists within surgery and will be reflected in our results, as surgery remains a male-dominated field despite observed changes in trainee gender distributions in recent years. This work also does not account for changes within the medical profession during the time period analysed, for example, the influence of the European Working Time Directive or other changes to training programmes. Bias may also have been introduced by limiting the search to the English language. This is perhaps reflected by the countries of study, with all taking place in the UK or USA, therefore, our findings may not necessarily be representative of surgeons from other countries. With any self-completed questionnaire, there may be self-selection bias as surgeons volunteered to participate in studies. Whilst validated personality tests were reported and met our inclusion criteria (Table 3), four out of six studies used different versions (short and long-form) of the Five Factor Model, with the remaining studies using other validated indices. We were unable to achieve our secondary outcomes (investigating post-operative outcomes in relation to surgeon personality).

## 5. Conclusions

There is increasing evidence that surgeon-specific factors influence decision-making. High levels of conscientiousness, openness and extraversion are commonly reported in abdominal surgeons, which contradict the ‘surgeon stereotype’ of rudeness and being difficult to work with. This manuscript sought to summarise the existing, variable quality of studies in an underexplored field, with the aim of informing future research on surgeon personality. Tentative evidence from this review would suggest that these personality traits may influence surgical decision-making, including the decision to anastomose, however, further work is necessary to confirm these findings. We suggest that for future research in this field, consensus usage of the Five Factor Model is necessary. Further clarification is necessary on the levels of emotional stability and agreeableness reported in surgeon populations. With wide variation in surgical practice reported by national datasets, greater understanding of how surgeon personality influences complex decision-making is necessary and may guide how surgeons respond to post-operative complications. The potential influence of personality on post-operative outcomes is yet to be established. Understanding the interaction between surgeon personality and cognitive bias bears implications for how we communicate management options with patients, and thus, the delivery of patient-centred care.

## Figures and Tables

**Table 1:** Data items.

Study Details	Title Year of Publication Authorship
Study Design	Cohort
	Case control
Participant Demographics	Specialty
	Experience
Validated Personality Index	Title of index used
Secondary Outcomes	Post-operative adverse events
Results	Raw data on personality scores
	Secondary outcomes

**Table 2:** Study characteristics.

Author	Year	Country	Experience	Number of Responders	Gender	Response Rate
Deary [26]	1992	UK	Trainees	22	5 Female	70%
					17 Male	
Drosdeck [27]	2015	USA	General Surgeons and Trainees	68	24 Female	45%
					44 Male	
McGreevy [28]	2002	USA	Trainees	39	15 Female	78%
					24 Male	
Moug [19]	2018	82% UK, 6% Ireland, 12% Other	Colorectal Surgeons	50	7 Female	N/A
					43 Male	
Shubeck [25]	2019	USA	Bariatric Surgeons	35	4 Female	N/A
					31 Male	
Whitaker [29]	2018	UK	General Surgeons and Trainees	172 (sub-group of 568 surgeons)	Unknown in sub-group	2.8%

**Table 3:** Study outcomes.

Author	Personality Tests Used	Surgeon Mean Personality Scores	Normal Population Comparison	Secondary Outcomes
Deary [26]	Eysenck Personality Questionnaire Edinburgh 6 Factor Personality Assessment	Not described	Not described	No
Drosdeck [27]	Big Five Inventory *	Agreeableness: 3.76 Conscientiousness: 4.3 Extraversion: 3.74 Neuroticism: 2.46 Openness: 3.75	3.13 [30] 3.73 3.82 3.82 3.9	No
McGreevy [28]	NEO-PI-R *	Male vs. Female Agreeableness: “average” Conscientiousness: 4.72 vs. 6.1 Extraversion: 3.27 vs. 6.05 Neuroticism: 2.35 vs. “average” Openness: 2.23 vs. 3.66	Male vs. Female *^†^* Agreeableness 47:46 Conscientiousness 58:63 Extraversion 57:63 Neuroticism 47:44 Openness 54:58	No
Moug [19]	Gosling Five Factor * Toronto Alexithymia Cognitive Reflex Thinking Type	Agreeableness: 4.9 Conscientiousness: 6.1 Emotional Stability: 5.4 Extraversion: 4.6 Openness: 5.4 Incidence of alexithymia: 4% Fast, intuitive thinking: 81%	3.74 3.65 2.97 3.24 3.67 13.4% [31] 10% *^††^* [32]	No
Shubeck [25]	Life Styles Inventory (LSI) ^§^	Aggressive: 41.7 (range 6–95) Constructive: 50.4 (13–97) Passive: 52.4 (14–94)	Not described in text	Yes (%) Post-op adverse event rate
Whitaker [29]	Big Five Inventory ^§^	Agreeableness: 76.3 Conscientiousness: 75.0 Extraversion: 54.7 Neuroticism: 60.6 Openness: 70.9	67.9 65.5 55.8 48.3 67.3	No

* Minimum Score 1, Maximum Score 5. ** Minimum Score 1, Maximum Score 7. ^§^ Minimum Score 0, Maximum Score 100. *^†^* Comparison with General Population. *^††^* Comparison with Medical Students.

**Table 4:** Newcastle-Ottawa scale for risk of bias assessment.

Author	Study Design	Selection (*max. 4*)	Comparability (*max. 2*)	Case-Control Exposure (*max. 4*)	Cohort Outcome (*max. 5*)	Risk of Bias *High/Med/Low*
Deary	Cohort	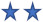	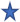	N/A	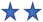	Medium
Drosdeck	Case Control	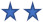	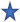	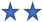	N/A	Medium
McGreevy	Cohort	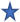	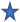	N/A	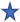	High
Moug	Cohort	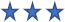	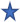	N/A	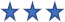	Low
Shubeck	Cohort	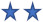	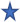	N/A	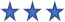	Low
Whitaker	Case Control	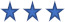	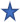	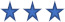	N/A	Low

## Supplementary Materials

The following are available online at https://www.mdpi.com/2076-328X/11/1/2/s1, Table S1.

## References

[1] Mayer J.D. (2017). Personality: A Systems Approach.

[2] Allemand M., Zimprich D., Hendriks A.A. (2008). Age differences in five personality domains across the life span. Dev. Psychol..

[3] Costa P.T., Terracciano A., McCrae R.R. (2001). Gender differences in personality traits across cultures: Robust and surprising findings. J. Personal. Soc. Psychol..

[4] Specht J., Egloff B., Schmukle S.C. (2011). Stability and change of personality across the life course: The impact of age and major life events on mean-level and rank-order stability of the Big Five. J. Personal. Soc. Psychol..

[5] Mittelstädt J.M., Pecena Y., Oubaid V., Maschke P. (2016). Psychometric personality differences between candidates in astronaut selection. Aerosp. Med. Hum. Perform..

[6] Sandal G.M., Endresen I.M., Vaernes R., Ursin H. (1999). Personality and coping strategies during submarine missions. Mil. Psychol..

[7] Chidester T.R., Helmreich R.L., Gregorich S.E., Geis C.E. (1991). Pilot personality and crew coordination: Implications for training and selection. Int. J. Aviat. Psychol..

[8] Sharma A., Sharp D.M., Walker L.G., Monson J.R. (2008). Patient personality predicts postoperative stay after colorectal cancer resection. Colorectal Dis..

[9] Weinryb R.M., Gustavsson J.P., Barber J.P. (1997). Personality predictors of dimensions of psychosocial adjustment after surgery. Psychosom. Med..

[10] Coker D.J., Koh C.E., Steffens D., Young J.M., Vuong K., Alchin L., Solomon M.J. (2020). The affect of personality traits and decision-making style on postoperative quality of life and distress in patients undergoing pelvic exenteration. Colorectal Dis..

[11] Schwartz R.W., Barclay J.R., Harrell P.L., Murphy A.E., Jarecky R.K., Donnelly M.B. (1994). Defining the surgical personality: A preliminary study. Surgery.

[12] Gilligan J.H., Treasure T., Watts C. (1996). Incorporating psychometric measures in selecting and developing surgeons. J. Manag. Med..

[13] Birkmeyer J.D., Reames B.N., McCulloch P., Carr A.J., Campbell W.B., Wennberg J.E. (2013). Understanding of regional variation in the use of surgery. Lancet.

[14] Pearse R.M., Harrison D.A., James P., Watson D., Hinds C., Rhodes A., Grounds R.M., Bennett E.D. (2006). Identification and characterisation of the high-risk surgical population in the United Kingdom. Crit Care.

[15] Neuman M.D., Bosk C.L. (2012). What we talk about when we talk about risk: Refining surgery’s hazards in medical thought. Milbank Q..

[16] Schwarze M.L., Barnato A.E., Rathouz P.J., Zhao Q., Neuman H.B., Winslow E.R., Kennedy G.D., Hu Y.Y., Dodgion C.M., Kwok A.C. (2015). Development of a list of high-risk operations for patients 65 years and older. JAMA Surg..

[17] Dekker S.W., Hugh T.B. (2008). Laparoscopic bile duct injury: Understanding the psychology and heuristics of the error. ANZ J. Surg..

[18] Sutherland F., Ball C.G. (2015). The Heuristics and Psychology of Bile Duct Injuries. Management of Benign Biliary Stenosis and Injury.

[19] Moug S.J., Henderson N., Tiernan J., Bisset C.N., Ferguson E., Harji D., Maxwell-Armstrong C., MacDermid E., Acheson A.G., Steele R.J. (2018). The colorectal surgeon’s personality may influence the rectal anastomotic decision. Colorectal Dis..

[20] Gosling S.D., Rentfrow P.J., Swann W.B. (2003). A very brief measure of the Big-Five personality domains. J. Res. Personal..

[21] Bisset C.N., McKee T., Tilling E., Cawley M., Moug S.J. (2020). Systematic review protocol examining the influence of surgeon personality on perioperative decision-making in abdominal surgery. BMJ Open.

[22] Moher D., Liberati A., Tetzlaff J., Altman D.G. (2009). The PRISMA Group. Preferred Reporting Items for Systematic Reviews and Meta-Analyses: The PRISMA Statement. PLoS Med..

[23] Wells G., Shea B., O’Connell D., Peterson J., Welch V., Losos M., Tugwell P. (2014). Newcastle-Ottawa Quality Assessment Scale Cohort Studies.

[24] Centre for Reviews and Dissemination, University of York (2009). Guidance: Systematic Reviews.

[25] Shubeck S.P., Kanters A.E., Dimick J.B. (2019). Surgeon leadership style and risk-adjusted patient outcomes. Surg. Endosc..

[26] Deary I.J., Graham K.S., Maran A.G. (1992). Relationships between surgical ability ratings and spatial abilities and personality. J. R. Coll. Surg. Edinb..

[27] Drosdeck J.M., Osayi S.N., Peterson L.A., Yu L., Ellison E.C., Muscarella P. (2015). Surgeon and nonsurgeon personalities at different career points. J. Surg. Res..

[28] McGreevy J., Wiebe D. (2002). A preliminary measurement of the surgical personality. Am. J. Surg..

[29] Whitaker M. (2017). The surgical personality: Does it exist?. Ann. R. Coll. Surg. Engl..

[30] Srivastava S., John O.P., Gosling S.D., Potter J. (2003). Development of personality in early and middle adulthood: Set like plaster or persistent change?. J. Personal. Soc. Psychol..

[31] Lane R.D., Lee S., Reidel R., Weldon V., Kaszniak A., Schwartz G.E. (1996). Impaired verbal and nonverbal emotion recognition in alexithymia. Psychosom. Med..

[32] Wen Tay S., Ryan P., Ryan C.A. (2016). Systems 1 and 2 thinking processes and cognitive reflection testing in medical students. Can. Med. Educ. J..

[33] Rentfrow P.J., Jokela M., Lamb M.E. (2015). Regional personality differences in Great Britain. PLoS ONE.

[34] Kirkham J.J., Altman D.G., Chan A.W., Gamble C., Dwan K.M., Williamson P.R. (2018). Outcome reporting bias in trials: A methodological approach for assessment and adjustment in systematic reviews. BMJ.

[35] Brand C.R., Egan V. (1989). The ‘Big Five’ dimensions of personality? Evidence from ipsative, adjectival self-attributions. Personal. Individ. Differ..

[36] Lovejoy C.A., Nashef S.A. (2018). Surgeons’ personalities and surgical outcomes. Bull. R. Coll. Surg. Eng..

[37] Quintero A.J., Segal L.S., King T.S., Black K.P. (2009). The personal interview: Assessing the potential for personality similarity to bias the selection of orthopaedic residents. Acad. Med..

[38] Bogacheva N., Kornilova T., Pavlova E. (2020). Relationships Between Medical Doctors’ Personality Traits and Their Professional Risk Perception. Behav. Sci..

[39] Logghe H.J., Rouse T., Beekley A., Aggarwal R. (2018). The evolving surgeon image. AMA J. Ethics.

[40] Bisset C.N., Dames N., Oliphant R., Alasadi A., Anderson D., Parson S., Cleland J., Moug S.J. (2020). Exploring shared surgical decision-making from the patient’s perspective: Is the personality of the surgeon important?. Colorectal Dis..

[41] Andrisano Ruggieri R., Iervolino A., Mossi P., Santoro E., Boccia G. (2020). Instability of Personality Traits of Teachers in Risk Conditions due to Work-Related Stress. Behav. Sci..

[42] Katz D., Blasius K., Isaak R., Lipps J., Kushelev M., Goldberg A., Fastman J., Marsh B., DeMaria S. (2019). Exposure to incivility hinders clinical performance in a simulated operative crisis. BMJ Qual. Saf..

[43] MacDermid E., Young C.J., Young J., Solomon M. (2014). Decision-making in rectal surgery. Colorectal Dis..

[44] Nicholson N., Soane E., Fenton-O’Creevy M., Willman P. (2005). Personality and domain-specific risk taking. J. Risk Res..

[45] Sharp G., Bourke L., Rickard M.J. (2020). Review of emotional intelligence in health care: An introduction to emotional intelligence for surgeons. ANZ J. Surg..

[46] Duckworth A., Gross J.J. (2014). Self-control and grit: Related but separable determinants of success. Curr. Dir. Psychol. Sci..

[47] Saposnik G., Redelmeier D., Ruff C.C., Tobler P.N. (2016). Cognitive biases associated with medical decisions: A systematic review. BMC Med. Inform. Decis. Mak..

[48] Furnham A., Marks J. (2013). Tolerance of ambiguity: A review of the recent literature. Psychology.

[49] Yee L.M., Liu L.Y., Grobman W.A. (2014). The relationship between obstetricians’ cognitive and affective traits and their patients’ delivery outcomes. Am. J. Obstet. Gynecol..

[50] Hearns S. (2019). Peak Performance Under Pressure.

[51] Brcic J. (2010). Motivational profile of astronauts at the International Space Station. Acta Astronaut..

[52] Epstein R.M., Hundert E.M. (2002). Defining and assessing professional competence. JAMA.

[53] Cornish J.A. (2020). Is being a role model a straightjacket or a privilege?. Bull. R. Coll. Surg. Eng..

